# Supplementary material for: Are carbon emissions trading and green financial instruments synergistic? -Comprehensive quantitative research based on content analysis
Source: PLoS One. 2024 Mar 7;19(3):e0298601. doi: 10.1371/journal.pone.0298601 (PMC10919601; doi:10.1371/journal.pone.0298601)
Supplement: S1 Data — (ZIP) [file pone.0298601.s001.zip › Data/地级市Z_F工作报告.zip!/─┌├╔╣┼/╬┌║ú╩╨/└┤╫╘╣1⁄2╓┌║┼í╛┬φ┐╦╩2╛▌═°í┐_2018.docx]

各位代表：

现在，我代表市人民政府向大会报告工作，请予审议，并请政协委员和其他列席人员提出意见。

一、过去五年和2017年工作回顾

五年来，我们在自治区党委、政府和市委的正确领导下，在市人大、政协的监督支持下，紧紧依靠全市各族人民，主动适应经济发展新常态，砥砺奋进，攻坚克难，经济社会保持了稳定发展态势，实现了“十二五”圆满收官、“十三五”良好开局，顺利完成市八届人大一次会议确定的各项工作任务，为全面建成小康社会奠定了坚实基础。预计地区生产总值年均增长8%，固定资产投资年均增长12.6%，社会消费品零售总额年均增长9.7%，城镇和农区常住居民人均可支配收入年均分别增长8.7%和4.4%。

五年来，我们坚持创新驱动、产业转型，发展动能不断汇聚。产业结构进一步优化。三次产业比重由0.9:68.2:30.9调整为1:60:39，规模以上工业增加值年均增长9.6%，第三产业增加值年均增长8%。工业转型步伐加快。煤焦化工、氯碱化工两大基地集群优势更加凸显，新能源、新材料、装备制造等战略性新兴产业占工业增加值比重达23%，高于全区平均水平。乌海市成为国家首批循环经济示范城市。服务业活力增强。万达广场、红星美凯龙等一批重点项目投入运营，消费结构不断升级。乌海湖获评国家级水利风景区，龙游湾湿地、金沙湾景区分别入选国家湿地公园、国家沙漠公园试点，新增4A级景区5家，旅游业收入年均增长25.8%，“大漠湖城”成为城市亮丽名片。金融机构达60家，存贷款余额均为五年前的1.4倍，金融业对GDP贡献率达到6.8%。特色农业发展迅速。“乌海葡萄”获地理标志证明商标，乌海市成为世界沙漠葡萄酒大赛永久举办地。“菜篮子”工程建设力度加大，建成便民蔬菜直销店43家。科技创新能力明显提升。全社会研发投入占比位居全区前列，建立智慧城市院士专家工作站，自治区级科技研发平台达到27个，国家高新技术企业达13家，乌海市荣获全国科技进步先进市。品牌战略深入实施。设立市长质量奖，中国驰名商标、内蒙古著名商标达26件。

五年来，我们坚持统筹兼顾、完善功能，城乡面貌大为改观。修订《乌海市城市总体规划》，编制完成《乌海市近期建设规划（2016—2020年）》和各类专项规划220余项。滨河一期建成，滨河二期稳步推进，建成区面积由40平方公里扩大到62.9平方公里，城市框架不断拉大。实施黄河海勃湾水利枢纽工程等一批重大基础设施建设项目，新建改造各类管网582公里，供热普及率、燃气普及率、生活垃圾无害化处理率分别达到92%、94%、99%。乌海湖大桥等一批重点桥梁道路建成通车，人均道路面积26平方米。乌海机场航线增至12条。开工建设棚户区改造等保障性住房5.2万套，4.2万户居民喜迁新居，乌海市被评为全国第七个住宅产业化[1]综合试点城市。建成绿地公园6个、人工湖29个，实施亮化项目308个，全市562个小区实现“五化”[2]全覆盖。城市精细化、智慧化管理水平不断提升，网格化管理模式基本形成，云计算中心投入使用，乌海市成为国家智慧城市、信息惠民城市试点，入选“宽带中国”示范城市。坚持创城为民、创城惠民、创城利民，扎实推进全国文明城市创建工作，切实补齐影响群众生活品质短板，市民文明素质和城市文明程度进一步提高。城乡一体化加快推进，完成农区危房改造6920户、街巷硬化396公里，综合执法、环卫保洁、文化惠民等基本公共服务向农区延伸。

五年来，我们坚持破立并举、改革开放，发展活力空前释放。供给侧结构性改革成果明显，化解煤炭过剩产能111万吨，消化房地产库存484万平方米，减免企业税费35.7亿元。“放管服”[3]改革不断深化，“双随机、一公开”监管制度全面推行。行政审批制度改革深入实施，非行政许可审批事项全部取消。“营改增”、不动产统一登记制度等改革任务全面完成。商事登记实现“二十证合一”[4]，市场主体从2.4万户增至4.1万户。区域协同发展实现破题开局，乌海及周边地区一体化[5]上升为自治区发展战略。对外开放水平持续提升，外贸进出口总额实现翻三番，与法国、蒙古国、意大利等国家友好城市合作进一步深化。

五年来，我们坚持生态优先、绿色发展，环境质量明显改善。区域大气污染联防联控力度加大，投入228亿元，组织实施大气污染治理项目2522个，焦化、电力、水泥行业污染防治取得初步成效，物料堆场实现全封闭，城区和工业园区20蒸吨以下燃煤锅炉全部淘汰。矿区环境综合整治扎实推进，煤矸石自燃得到有效治理，中心城区空气质量优良天数比五年前增加110天。主要污染物减排工作成效显著，乌海市荣获自治区二氧化硫减排和氨氮减排突出贡献奖。《乌海市海勃湾生态涵养区保护条例》颁布实施，水源地水质达标率100%。生态建设力度不断加大，投入是上个五年的2.7倍，实施绿化项目117个，建成区绿化覆盖率由38%提高到43%，人均公园绿地面积19.9平方米，乌海市跨入全国绿化模范城市和国家园林城市行列。

五年来，我们坚持民生为本、加大投入，人民幸福感持续提升。民生和社会事业累计支出371亿元，占财政支出的70%以上。就业创业工作稳步推进，城镇新增就业4.2万人，城镇登记失业率控制在自治区下达目标任务范围内，3家企业入围国家级众创空间。社会保障扩面提标，退休人员养老金待遇连续十三年增长，低保标准等主要民生指标居全区前列，乌海市入选全国首批全民参保登记计划试点城市、第二批电子社保示范城市。脱贫攻坚工作成效显著，1434名贫困人口稳定脱贫。养老服务体系进一步完善，建成老年公寓、社区老年人日间照料中心和农区养老服务中心48个。教育事业稳步发展，新建扩建幼儿园37所，三区全部通过国家义务教育基本均衡县验收，各层次教育发展步伐加快。医药卫生体制改革深入推进，公立医院综合改革受到国务院表扬。文化事业繁荣发展，当代中国书法艺术馆等一批公共文化服务设施建成使用，“书法五进”[6]入选国家公共文化服务体系示范项目，黄河明珠·中国乌海书法艺术节品牌影响力持续提升。乌海市荣获全国围棋之乡称号。足球改革试点取得初步成效，学校体育场馆免费向市民开放，2名乌海籍运动员荣获世锦赛冠军。实施公交票价减免政策，投放新能源公交车80辆，共享单车广泛普及，绿色出行成为新风尚。连续七届入选自治区双拥模范城，进入全国双拥模范城行列，乌海市被授予全国无障碍先进示范市、全国残疾人工作先进单位。

五年来，我们坚持转变作风、提升效能，政府自身建设全面加强。深入学习贯彻党的十八大、十九大精神，扎实开展党的群众路线教育实践活动和“三严三实”专题教育，推进“两学一做”学习教育常态化、制度化，政府作风持续转变。依法向人大及其常委会报告工作，向政协通报工作情况，办理人大建议、政协提案1384件，满意率98%。依法行政深入推进，法律顾问制度普遍建立，“六五”普法圆满完成，“七五”普法全面实施。完成新一轮政府职能转变和机构改革，行政权力运行更加规范。严格执行中央八项规定和国务院“约法三章”，公务用车制度改革、办公用房清理圆满完成，“三公”经费支出年均下降28.8%，“四风”问题得到有效遏制。反腐败斗争不断深入，418个违纪违法问题得到严肃查办。

各位代表，刚刚过去的2017年，是本届政府的收官之年。我们认真贯彻落实中央、自治区各项工作部署，扭住发展不放松、攻坚克难不退缩，全力抓重点、补短板、强弱项，各项工作取得了新进展。

发展基础更加坚实。把项目建设作为发展第一动力，深入实施“重点项目建设年”活动，建立领导干部包联项目责任制，215个重点项目开工建设，其中10亿元以上项目24个，工业固定资产投资增长63%。非公有制经济快速发展，民间投资增长40%，为经济可持续发展提供了有力支撑。一系列稳增长政策举措落地见效，建立5000万元助保贷资金池，帮助企业转续贷155亿元、倒贷68亿元。通过电力多边交易降低企业用电成本4.7亿元。融资渠道不断拓宽，世环新材料登陆“新三板”，6家企业在内蒙古股权交易中心挂牌。一批政府和社会资本合作项目顺利实施，海勃湾区PPP模式受到国务院表彰。招商引资力度加大，全年签约项目43个，到位资金135亿元，同比增长10.9%。

发展动力持续增强。坚持转型发展，积极培育接续替代产业，形成多点支撑、多轮驱动发展格局。在中央媒体测评中，乌海市经济转型能力居全区首位。工业经济焕发新活力。传统产业向清洁化延伸，家景镁业30万吨甲醇、嘉盛焦粒制气项目投产，源通焦炉煤气制LNG项目试生产，洪远聚乙烯多联产示范项目开工建设。精细化工向高端化迈进，黑猫特种炭黑投产，乌海化工特种PVC项目主体建成，卡博特恒业成气相二氧化硅、源宏医药中间体二期进展顺利，永宁医药原药等项目签约落地。新兴产业向规模化发展，陕汽新能源汽车产量突破6000辆，中钰镁合金轮毂及板材项目扎实推进，京运通硅材料、宝武集团炭材料、英能100兆瓦光伏电站等项目开工建设。军民融合战略稳步实施，航天十二院氢等离子体煤制乙炔项目[7]顺利推进，建成军民融合产业数据库，成功举办第九期“钱学森论坛”。服务业呈现新气象。乌海湖一号码头及游客服务中心投入使用，甘德尔山生态文明景区、满巴拉僧庙景区基本建成，成吉思汗博物馆主体完工。全年接待游客293万人次，实现旅游收入63亿元，分别增长39%、57%，乌海市获评2017年中国魅力城市，城市美誉度进一步提升。乌海海关通关，国际陆港、唐山港·乌海内陆港运营，乌蒙欧国际班列通车，乌海及周边地区货物通关实现“一站式”服务。人民币存、贷款余额预计分别达到797亿元、613亿元，同比增长13.4%、14.8%，金融业税收贡献率达11.2%。特色农业得到新提升。“丝绸之路”世界沙漠葡萄酒文化节成功举办，乌海市被认定为“世界沙漠绿洲葡萄酒产区”。完成全市永久基本农田划定工作。完善农企利益联结机制，带动2万余名农区居民增收致富，绿色生态农业产业园入选全国首批创业创新园区。

发展短板加快补齐。编制完成《七大基础设施网络体系建设规划》，基础设施短板进一步补齐。交通体系更加健全。完成沃野路、狮城街等48条城区道路新建改建，打通“断头路”11条，改造人行道14.3万平方米。荣乌高速乌海段、客运枢纽站一期主体完工，国道110线黄河特大桥等项目顺利实施，外环内网交通格局基本形成。市政设施更加便民。餐厨垃圾资源化利用和无害化处理项目投入运营，海勃湾南部净水厂实现供水。完成既有建筑节能改造93.5万平方米，改造各类管网80余公里，增设停车位2.3万个，新建改建公厕41个。新型智慧城市[8]加快建设，智慧公交投入运营，乌海市被列入国家“互联网+政务服务”示范工程名单。园区配套更加完善。实施工业园区基础设施项目18个，低碳产业园西环路、乌达工业园蒸汽管网投入使用，海南工业园综合管廊一期等项目开工建设，海勃湾工业园供水工程等项目加速推进。海南工业园增量配电入选国家试点。

发展难题积极破解。坚持问题导向，定向施策，精准发力，着力解决制约发展的重点难点问题。破解改革攻坚难题。物流业改革创新试点等7项国家、自治区改革试点任务稳步推进，社会信用体系建设改革等5项试点任务顺利完成。农区综合改革不断深化，海南区土地承包经营权确权登记颁证试点取得阶段性成果。按照自治区统一部署，制定规范政府债务管理、防范金融风险方案，坚决叫停过度超前、盲目融资举债项目，财政金融风险得到有效防控。破解环境治理难题。以中央环保督察和自治区生态环保大检查反馈意见整改为契机，实施生态建设和环境保护等“十大行动”[9]，一批突出环境问题得到整改落实。组织实施大气污染治理项目347个，电力企业超低排放改造有序推进，完成矸石、煤层自燃火点治理121处、66万平方米。区域大气污染联防联控力度不断加大，全年中心城区空气质量优良天数达到268天。实施甘德尔山生态综合治理二期、矿山植被恢复试点等生态建设项目22个，完成造林绿化面积2.5万亩。“河长制”[10]责任体系全面建立，乌海市获评全国首批水生态文明城市。破解区域协作难题。编制完成《乌海及周边地区一体化发展规划纲要》，黄河金岸蒙西四盟市[11]区域发展协作机制进一步完善，区域检验检测平台、农产品销售网点等24个合作项目顺利推进，乌海及周边地区一体化取得实质性进展。主动与周边地区联络对接，成功举办蒙宁陕甘、呼包银榆等区域协作发展会议。破解服务效能难题。成立行政审批服务局，市民服务中心建成启用。推行重大项目并联审批，实现全程网上办理，审批时限压缩50%以上。“八张清单”[12]改革深入推进，实现法无授权不可为、法定职责必须为。

发展成果全民共享。坚持以人民为中心的发展思想，一大批惠民举措落地见效，群众获得感显著增强。围绕贫有所助，认真落实“六个精准”“五个一批”要求，建立贫困人口动态管理机制，年度脱贫任务圆满完成。围绕住有所居，实施棚户区改造项目11个，开工12637套，建成4840套，7000户居民搬迁入住。围绕劳有所得，扎实推进就业创业，新增城镇就业7741人，城镇登记失业率3.46%。依法维护劳动者权益，清欠农民工工资6300万元。城镇和农区常住居民人均可支配收入分别达到39436元、16790元，增长8%、8.5%。围绕弱有所扶，不断提高社会兜底保障水平，低保标准由585元提高到635元，特困供养人员集中、分散生活补贴标准全年增加1200元，居自治区前列。围绕老有所养，加快完善养老服务体系，市老年养护中心等3所养老机构主体建成，城乡养老服务覆盖率高于全区平均水平。围绕学有所教，新建幼儿园3所，学前三年毛入园率95%，普通高考本科上线率78.9%，义务教育、民族教育、特殊教育、职业教育和高等教育稳步发展。围绕病有所医，深入推进公立医院综合改革，药品售价平均下降13%。医保报销实现区内、跨省异地就医直接结算，居民医保财政补助标准居全区首位。围绕民有所乐，举行广场文化艺术节、全民歌咏大会等文化惠民品牌活动540余场次，成功举办环乌海湖国际马拉松等体育赛事，文化体育事业蓬勃发展。围绕食有所安，食品药品监管所标准化改造全部完成，新建食品快检室21个，“舌尖上的安全”得到有效保障。侯卫东官场笔记围绕治有所为，开展社会矛盾积案化解“百日攻坚”行动，一批信访积案妥善化解。“安全生产年”各项工作深入推进，信息化监管平台建成使用，安全生产形势总体稳定。深化平安乌海建设，立体化治安防控体系不断完善，人民群众安全感和满意度显著提升。成功举办庆祝自治区成立70周年系列活动。支持工会、共青团、妇联等人民团体和各类社会组织开展工作，国防建设、民族宗教、计划生育、外事侨务、人防气象、防震减灾、档案史志、新闻出版、广播影视、无线电管理、红十字会、残疾人等各项工作取得新成绩。

各位代表，过去五年的成绩来之不易。这是党中央、国务院和自治区党委、政府及市委坚强领导的结果，是市人大、市政协大力支持、有效监督的结果，是广大干部群众团结一致、顽强拼搏的结果。在此，我代表市人民政府，向各民主党派、工商联、人民团体、驻市单位、驻市部队以及社会各界人士，致以崇高的敬意和衷心的感谢！

五年的发展实践给予我们深刻启示，是我们不忘初心、继续前进的宝贵财富。一是必须牢牢把握正确政治方向这一根本原则。牢固树立政治意识、大局意识、核心意识、看齐意识，不折不扣、坚决有力贯彻落实中央、自治区党委和市委各项决策部署。二是必须牢牢把握转型发展这一首要任务。坚持新发展理念，以提高发展质量和加快转变经济发展方式，推动经济实现更高质量发展。三是必须牢牢把握改革创新这一核心动力。拿出“敢为天下先”的魄力，突出重点、对准焦距、击中要害，着力破除各类体制机制障碍，充分释放发展活力。四是必须牢牢把握生态环保这一基础底线。以对党和人民、对子孙后代高度负责的精神，做好生态建设和环境保护工作，促进生态与经济协调发展。五是必须牢牢把握改善民生这一根本目的。积极回应人民群众对美好生活的向往，用心用情用力抓好民生工作，不断提升人民群众幸福指数。六是必须牢牢把握担当实干这一基本要求。紧紧围绕资源型城市转型这一目标，勤政务实、勇于开拓，咬定青山不放松，一张蓝图干到底。

需要向代表说明的是，受宏观经济下行、产业结构调整、网络消费外流等因素影响，2017年乌海市地区生产总值、固定资产投资、社会消费品零售总额等指标增长低于预期目标。针对多年积累的财政收入空转虚增问题，我们按照自治区统一部署，下决心坚决纠正，调减了一般公共预算收入任务，财政收入指标大幅度下降。但总体来看，经济发展质量和效益明显提高，核心经济指标增速位居全区前列。同时，我们也清醒地看到，经济社会发展不平衡不充分问题仍然突出：一是产业结构深度调整任务艰巨，带动性强的大项目、好项目不多，固定资产投资乏力，经济增长后劲不足。二是资源环境约束趋紧，矿区环境综合整治、大气污染联防联控等环境治理任务十分艰巨。三是基础设施还存在短板，区域中心辐射带动力不强，城市精细化管理水平有待提升。四是民生保障仍需加强，教育、医疗等社会事业发展与群众对美好生活的向往仍有差距。五是对标全面建成小康社会指标体系，环境质量、服务业发展等方面指标实现程度还不高。六是政府职能转变还不到位，部分干部不善为、不能为、不作为现象依然存在。对此，我们必须增强忧患意识和担当精神，不回避、不推诿、不懈怠，精准施策，持续用力，推动政府各项工作再上新台阶。

二、今后五年目标任务

今后五年，是我国“两个一百年”奋斗目标的历史交汇期，也是乌海市转型发展的关键期。在这样一个重要历史时期，需要我们在国家和自治区发展大局中寻找乌海的位置、谋划乌海的未来。从宏观层面看，世界经济进入深度转型调整期，新一轮科技革命和产业革命正在兴起，对经济发展产生了重大影响。我国经济韧性好、潜力足、回旋余地大，长期向好的基本面没有变，为乌海市转型发展提供了难得机遇。从自治区层面看，我区资源禀赋、区位条件、要素成本等多重优势充分发挥，自治区打造国家重要能源基地、新型化工基地、战略性新兴产业基地的发展定位，以及推进中蒙俄经济走廊和沿黄经济带建设，为乌海市转型发展提供了有利条件。从乌海市层面看，经济结构调整和动能转换加快推进，传统产业焕发出新活力，新兴产业持续壮大，新的经济增长点不断涌现，党的十九大明确支持资源型地区经济转型发展，乌海发展站在了一个新的历史起点上。我们要以更大的决心、更强的力度、更实的举措，全力以赴开创乌海转型发展新局面。

今后五年政府工作的总体要求是：深入贯彻落实党的十九大精神，以习近平新时代中国特色社会主义思想为指导，统筹推进“五位一体”总体布局，协调推进“四个全面”战略布局，全面落实自治区党委、政府和市委决策部署，把握稳中求进工作总基调，坚持新发展理念，以推进供给侧结构性改革为主线，推动新型工业化、信息化、城镇化、农业现代化同步发展，打好防范化解重大风险、精准脱贫、污染防治三大攻坚战。以打造区域创新型城市为统领，牢牢把握高质量发展根本要求，坚持“两个着力”，加快“五区”建设，如期全面建成小康社会，以优异成绩迎接建党一百周年。

今后五年经济社会发展主要目标是：区域创新型城市稳步推进，经济转型和城市转型取得新成效，发展质量效益明显提高，人民生活品质大幅提升。2020年，与全国同步全面建成小康社会，圆满完成“十三五”发展目标任务。2022年，自治区西部区域中心地位更加凸显，辐射带动作用显著增强，实现更高质量发展。

——实施产业升级战略，打造转型示范之城。坚持做大总量与做优结构并重，加快经济由工业主导型向多元驱动型转变，推动乌海经济提档升级。坚定不移推进产业结构调整和发展方式转变，推动传统产业向新型化、高端化发展，不断培育壮大战略性新兴产业，提升现代服务业和特色农业发展规模和层次，构建特色鲜明的现代化经济体系。推进与周边地区资源共享、产业链接、园区循环，培育形成自治区西部新的经济增长极，着力打造转型发展示范区。

——实施创新驱动战略，打造创新创业之城。坚持创新第一动力，建立以政府为主导、企业为主体、产学研深度融合的科技创新体系。依托乌海市新型研发机构和企业研发中心，深化与国内高等院校和科研院所合作，围绕产业互促和军民融合等重点领域攻克一批关键技术，打造自治区西部区域创新中心。实施人才强市战略，探索柔性人才引进机制，积极引进高层次和技能型人才，营造识才爱才敬才用才的良好氛围。依托煤焦化工、氯碱化工产业优势和职业教育基础，打造区域高技能人才高地。进一步完善政策、搭建平台，激发广大青年创新创业热情，形成全社会追求梦想、干事创业的生动局面。

——实施品质提升战略，打造宜居宜游之城。深化产城融合，完善功能品质，推动城市发展由外延扩张式向内涵提升式转变。按照“提升老城区、繁荣新城区、改造棚户区”的思路，统筹城市规划建设管理，完善“七网”[13]公共基础设施，着力打造宜居城市环境。充分发挥大河、大湖、大漠、大汗、大湿地等自然景观优势和黄河文化、蒙元文化、书法文化等独特文化优势，推动文化与旅游产业深度融合，打响“大漠湖城”旅游品牌。统筹推进城乡一体化发展，着力打造城乡融合先行区。

——实施开放带动战略，打造魅力活力之城。构建开放大通道，依托乌海区位优势，加快交通网络和枢纽节点建设，提升对外开放通达能力。搭建开放大平台，积极融入“一带一路”建设和“中蒙俄经济走廊”，充分发挥海关、国际陆港、内陆港作用，大力发展跨境贸易、跨境电子商务，着力打造区域对外开放新高地。推动开放大协作，加快乌海及周边地区一体化进程，促进区域人流、物流、资金流、信息流集中集聚，着力打造环境最优、活力最强的投资贸易首选区，成为服务周边地区的“加油站”和“后花园”。

——实施绿水青山战略，打造美丽生态之城。践行“绿水青山就是金山银山”的理念，像保护眼睛一样保护生态环境，像对待生命一样对待生态环境。推进大气污染联防联控，全面落实“河长制”，下大力气解决突出环境问题，坚决打赢蓝天保卫战。实施生态系统保护和修复工程，编织覆盖城乡的绿色网络体系，着力打造生态环保攻坚区。

——实施民生优先战略，打造文明幸福之城。顺应人民群众对美好生活的新期待，不断加大民生投入，持续增加居民收入，让发展成果更多更公平地惠及全市人民。培育和践行社会主义核心价值观，扎实推进全国文明城市创建工作，全面提升两个文明建设水平。统筹解决好脱贫攻坚、就业创业、社会保障、棚户区改造、食品药品安全等方面问题，不断增强人民群众的获得感、幸福感和安全感。加快补齐教育、医疗等民生短板，打造优教优医普惠区，大幅提升城市综合竞争软实力。

三、2018年工作安排

2018年是贯彻党的十九大精神的开局之年，是改革开放40周年，是决胜全面建成小康社会、实施“十三五”规划承上启下的关键一年。能否实现顺利开局，直接关系未来五年乌海的发展。我们要精准聚焦市委提出的主攻方向，始终保持勇立潮头的闯劲，始终保持攻坚克难的韧劲，始终保持为民造福的干劲，全力以赴狠抓落实，朝着决胜全面建成小康社会目标奋勇前进。2018年的主要预期目标是：地区生产总值增长6.5%左右，城乡居民人均可支配收入分别增长7%左右和7.5%左右，城镇新增就业7500人以上，城镇调查失业率和城镇登记失业率控制在自治区下达目标以内；居民消费价格涨幅控制在自治区调控目标以内；全面完成节能减排目标任务；供给侧结构性改革取得实质性进展；政府债务化解完成年度任务，财政金融风险有效防控。对一般公共预算收入、固定资产投资、社会消费品零售总额指标，分别按6%、9%、7.5%掌握，以支撑高质量发展要求。

围绕以上目标，2018年，乌海市将着力做好以下七方面工作：

（一）聚焦项目建设，着力增强经济发展动力

坚持经济工作项目化、项目建设清单化，狠抓项目推进，推动经济持续健康发展。加速项目建设。全年计划实施重点项目175个，完成投资246亿元。继续实行领导干部包联重点项目责任制，倒排工期、挂图作战。加强经济运行监测分析，科学研判、定期调度，确保经济稳定增长。强化服务保障。推进“互联网+政务服务”，建立电子证照库，实现数据共享，让信息多跑路、企业少跑腿。优化营商环境，充分激发市场主体活力。鼓励金融机构增加信贷投放，缓解企业资金困难。落实援企稳岗、减税降费、增量配电、电力多边交易等政策措施，降低企业运营成本。精准招商引资。采取专业化集群式招商方式，积极承接京津冀、环渤海、长三角等地区产业转移。围绕非煤非资源型产业、文化旅游产业等领域，引进一批优质项目。做好重点项目论证，积极争取国家、自治区政策和资金支持。完善园区配套。推进“多规合一”，完善《乌海经济开发区总体规划》，统筹推进“一区四园”建设，探索工业园区“园中园”模式，实现园区特色化、差异化发展。加大园区基础设施投入，补齐低碳产业园和工业园区基础配套设施短板，提升园区承载能力。盘活工业用地资源，综合运用市场机制和法治办法，多措并举做好长期停产企业土地收储工作，逐步实现“腾笼换鸟”。加大全方位服务企业力度，切实提升园区“软实力”。

（二）聚焦产业转型，着力构建现代化经济体系

坚持“经济转型、动能转换”双轮驱动，不断优化经济结构，破除对资源型产业的路径依赖，构建多元发展、多极支撑的现代化经济体系。

实施创新驱动发展工程。持续加大科技投入，围绕煤焦化工、氯碱化工产业链布局创新链，推进政产学研用协同创新，加快科技成果转化。积极培育国家级高新技术企业，推进国家级高新技术产业开发区创建工作。深入实施军民融合发展战略，编制《乌海市军民融合深度发展规划》，创建军民融合产业化中心，推进氢等离子体煤制乙炔项目取得实质性进展。

实施传统产业升级工程。编制《乌海市产业结构转型升级规划》，以产品创新升级为突破点，推动传统产业迈向中高端。抓住国家“去产能”政策机遇，切实盘活存量、做大增量。围绕优势产业延链补链，加快洪远聚乙烯、东源1,4—丁二醇二期、安泰蒽油加氢等项目建设，促进信诺立兴煤清洁高效综合利用项目落地。推动华信LNG等项目投产，积极争取蒙西煤制天然气外送通道、鄂尔多斯—安平—沧州输气管道延伸接入乌海。引导精细化工集聚发展，加快卡博特恒业成气相二氧化硅、中瑞化工等项目建设，确保亚东萘系列产品、源宏医药中间体二期等项目建成投产。

实施新兴产业提速工程。编制《战略性新兴产业发展规划》，培育壮大经济发展新动能。着力打造新能源产业高地，推动阿特斯光伏、五凌光伏等项目并网发电，力争东方日升二期、润泰绿色智慧能源多联供项目开工。编制《充电基础设施规划》，开展电动公交车试点，逐步降低燃油、天然气公交车比例。健全新材料产业体系，抓好中国宝武集团炭材料、泰和玻璃纤维等项目建设，确保京运通硅材料项目投产。大力发展装备制造业，推动上海荷福飞行器和智能机器人项目落地，促进陕汽新能源汽车增产扩能，力争中钰镁合金轮毂及板材项目建成。

实施现代服务业提质工程。制定物流业减费增效政策措施，抓好乌海北货场、内蒙古西部智慧物流港、神华煤炭物流中转基地等物流园区建设。提高金融服务实体经济能力，完成乌海银行增资扩股。推动恒业成有机硅等企业在主板上市，支持企业签约新三板。加大浙商国际商贸城、金裕二期等项目建设力度，提升商贸服务、餐饮住宿、体育休闲、健康养老等生活性服务业发展质量和层次，带动区域消费需求。创新住房公积金管理制度，激活住房等大额消费活力。推进信息产业发展，加快区块链大数据产业园等项目建设，改造升级创客空间，着力打造自治区西部区域信息中心。

实施特色农业提升工程。深入实施乡村振兴战略，推进农业供给侧结构性改革，构建以葡萄和葡萄酒产业为核心的现代农业产业体系。健全农企利益联结机制，鼓励汉森、阳光田宇等农业龙头企业加快发展，打响世界沙漠葡萄酒品牌。大力发展设施农业，切实保障市民“菜篮子”。依托蒙根花光伏农业园、绿色生态产业园等农业综合体，培育发展乡村休闲旅游，促进三次产业深度融合。稳妥推进土地草牧场所有权、承包权、经营权“三权”分置改革，推进土地和草牧场确权颁证工作，促进土地有序流转，适度发展农区集体经济。深入推进城乡一体化，加大农区环境综合整治力度，提升农区基础设施建设和公共服务水平。

（三）聚焦城市建设，着力提升滨水城市品位

立足自治区西部区域中心定位，切实做好“水”“城”“绿”三篇文章，打造美丽乌海第一印象。

以水为魂，增添城市灵气。完善码头、停车场等旅游基础设施，加快高山草甸、乌海院子等项目建设，积极创建乌海湖国家休闲旅游度假区，支持海勃湾区创建国家级全域旅游示范区。深入实施“文旅+”发展战略，加快甘德尔山蒙元文化产业园等文化旅游项目建设，开展水上运动、沙漠越野、低空飞行等“海陆空”多维度体验项目，开发冰雪嘉年华等冬季旅游项目，丰富旅游产品体系。加快推进河道综合整治，切实改善城市水系环境。深化与中青旅等国内一流旅游运营商合作，创新旅游营销模式，拓展文化内涵，讲好“乌海故事”，树立乌海旅游品牌形象。推出精品酒庄、黄河风情、驼盐古道等旅游线路，完善吃住行、游购娱“一条龙”配套服务，力争接待游客突破350万人次，实现旅游收入突破75亿元。

以城为心，激发城市活力。牢固树立“大城建”理念，以城市总体规划为蓝图，全面落实主体功能区规划，探索推进“多规合一”。以城中棚户区、东山棚户区为重点，加大棚户区搬迁改造力度，推行货币化安置，完成14个棚户区改造项目、8830套保障性住房建设任务。以全国文明城市创建为重要抓手，加强市政设施建设和城市精细化管理，大力整治背街小巷、城乡结合部等城市建设管理死角，加快老旧小区供热管网、城区燃气管网、给排水管网改造，补齐城市建设短板。按照国家、自治区统一部署，稳步推进住房制度改革，促进房地产市场平稳健康发展。完善城市路网体系，推动包银高铁乌海段、省道S217线海勃湾绕城段及连接线项目开工建设，启动海南区至鄂尔多斯棋盘井快速通道、甘德尔公铁立交桥工程和乌海客运枢纽站二期，新建改建东环路等城区道路41条，加快乌达巴音赛互通立交桥建设，建成国道110线黄河特大桥，实现荣乌高速乌海段全线通车。新开通乌海至青岛、乌鲁木齐等航线，提高城市通达能力。实施饮水安全工程，加快海勃湾北部净水厂建设，建成乌达和海南净水厂。推进甘德尔山体、乌海湖大桥、滨水岸线等重点区域、重要节点景观亮化，着力打造魅力夜景城市。深入推进“厕所革命”，新建改建旅游厕所20个。整合市政公用、市容环卫、园林绿化等城市管理职能，提高城市综合执法效能。本着“尊重历史、实事求是”的原则，加快解决人民群众普遍关心的房产证办理遗留问题。完善物业管理体制机制，下大力气提升物业服务水平。统筹城乡发展，因地制宜打造一批产业特色鲜明、绿色生态宜居、文化旅游融合的精品特色小镇。

以绿为脉，擦亮城市底色。大力推进京津风沙源治理、天然林保护等国家重点林业工程，加快乌海湖两岸、甘德尔河岸等园林绿化项目建设，构建绿色城市生态体系。加大龙游湾湿地公园建设保护力度，积极探索林木林地保护管理新机制。通过见缝插绿、腾地换绿等方式，新建一批公共休闲绿地公园，推进荣乌高速乌海段等国省干道、城际快速通道沿线绿化美化，大幅提升城市总体绿量，让绿色成为城市版图主色调。

（四）聚焦改革开放，着力培育城市发展新动能

把深化改革开放作为发展的“关键一招”，再创体制机制新优势、对外开放新格局。全面深化重点领域改革。深化供给侧结构性改革，破立结合推动实体经济提质增效。推进“放管服”改革，精简审批服务环节、优化审批服务流程，实现“一号”申请、“一窗”受理、“一网”通办[14]，着力打造审批事项最少、时限最短、服务最优城市。完成相对集中行政许可权改革，完善权力清单、责任清单、市场准入负面清单，以清单管理推动减权放权。强化投融资体制改革，清理废除妨碍公平竞争的各种规定和做法，鼓励社会资本参与基础设施等重点项目建设。推进财政体制改革，合理界定市区两级财政事权和支出责任。强化财政收入预算管理，严格依法依规组织财政收入。完善诚信体系，打造自然人信用信息库，实行失信行为联合惩戒机制。纵深推进对外开放合作。认真落实《乌海及周边地区一体化发展规划纲要》，确保区域产业共育、基础设施共建、旅游资源共享等领域合作有序推进。积极协调周边地区，推进乌海西—吉兰泰—瓦窑—策克铁路、东乌铁路和蒙宁5城11园铁路环线前期工作。鼓励企业在策克、乌力吉、甘其毛道口岸设立物流中心，打造区域煤炭资源储备加工基地。推行海关一次申报、一次检验、一次放行联合监管模式，促进贸易便利化。设立口岸保税仓，强化与“一带一路”沿线国家贸易往来，力争进出口贸易总额增长10%以上。拓展国际城市“朋友圈”，在文化旅游、医疗卫生等领域打造更多合作亮点。

（五）聚焦环境治理，着力促进人与自然和谐共生

坚持绿色发展理念，切实解决环保突出问题，让天更蓝水更绿空气更清新。强化大气污染防治。编制《城市修补生态修复专项规划》，实施大气环境综合整治项目93个，大力推进电力企业超低排放改造，严格控制扬尘等面源污染，积极开展二氧化硫污染专项整治，全面淘汰矿区20蒸吨及以下燃煤锅炉。加强乌海及周边地区大气污染联防联控，建立联合执法机制，零容忍查处环境违法行为。积极推进第三批国家低碳城市试点，力争单位GDP能耗下降3.1%，空气质量优良天数达到280天以上。推进矿区环境综合整治。实施综合整治项目41个，完成排土场整治6处、治理煤层自燃7处。完善矿山企业生态环境准入退出机制，建立矿山地质环境治理恢复基金，压紧压实企业主体责任。全力打造绿色矿山，探索建设矿山地质公园，确保矿区环境明显好转。加大水污染治理力度。实行最严格水资源管理制度，严格控制地下水超采。全面推行“河长制”，强化黄河水体保护，推进乌海湖清淤和资源化利用，加强库区冬季扬尘治理。大力整治生活污水散排问题，城镇污水处理达到国家排放标准。加强土壤污染防治。严格落实耕地保护和节约集约用地制度，启动第三次全国土地调查，实施土地资源消耗和强度“双控”行动。深化生态文明体制改革。推行领导干部自然资源资产离任审计，落实环境污染责任终身追究制。探索市场化、多元化补偿机制，确保西鄂尔多斯自然保护核心区乌海境内企业如期退出。

（六）聚焦民生保障，着力满足群众对美好生活的需要

坚守底线、突出重点、完善制度、引导预期，扩大和优化公共服务供给，进一步提升人民生活水平。实施精准脱贫计划。坚持精准识别、精准帮扶，打好健康扶贫、产业扶贫、教育扶贫等“组合拳”，激发贫困人口内生动力。健全脱贫监测评估和防范返贫机制，做好脱贫人员后续帮扶和巩固提升工作，确保高质量稳定脱贫。实施创业增收计划。推进大众创业、万众创新，加强众创空间和创业孵化基地建设。统筹抓好高校毕业生、复转军人、就业困难人员等重点群体就业创业，保持零就业家庭动态清零，城镇登记失业率控制在自治区下达目标以内。实施社会保障计划。健全社会保障体系，加快电子社保示范城市建设，不断扩大“五险”覆盖面。构建多元化社会救助体系，推动基本医保、大病保险和医疗救助有效衔接。以推进国家级医养结合试点为契机，鼓励社会力量兴办养老机构，进一步健全养老服务体系。实施教育提质计划。启动第三期学前教育行动计划，加大公办幼儿园建设力度，积极探索民办公助模式，切实缓解适龄幼儿“入园难”问题。促进教育优质均衡发展，提高义务教育、普通高中教育教学质量，打造一批名师名校。完善职业教育和培训体系，启动职业技能公共实训中心二期建设。稳步发展民族教育、特殊教育、高等教育。实施健康乌海计划。深化医疗卫生体制改革，深入实施医疗、医保、医药“三医联动”，完善分级诊疗制度。提升医疗卫生技术水平，打造一批名医名院。开工建设人民医院新院，着力创建三甲医院。加快蒙中医院二期建设，推动蒙中医药产业发展。深入开展爱国卫生运动，积极创建国家卫生城市。完善“全面两孩”配套政策，促进人口长期均衡发展。实施文体惠民计划。完善现代公共文化服务体系，大力弘扬乌兰牧骑精神，深入开展文化惠民活动。办好第十届黄河明珠·中国乌海书法艺术节暨书法产业博览会，发展教育培训、展览展销、评估鉴定、拍卖交易等书法产业，提升中国书法城品牌形象。加强召烧沟岩画、古长城等文化遗产保护利用。抓好内蒙古西部足球训练基地建设，举办环山湖自行车、沙漠越野等重大赛事活动。实施重大风险防范计划。加强政府债务风险管理，全力化解政府债务，坚决守住不发生系统性金融风险底线。强化矛盾纠纷排查化解，畅通民情民意诉求渠道。完善安全生产责任体系，深入开展安全生产标准化、智慧化建设，坚决遏制重特大安全生产事故。健全食品药品监管信息平台，建立农产品质量安全追溯体系，扎实推进国家食品安全城市创建。整合检验检测资源，打造区域检验检测中心。推进警务大数据平台建设，提高社会治安防范能力，确保社会和谐稳定。

做好“十三五”中期评估。开展“七五”普法中期验收。充分发挥工会、共青团、妇联等人民团体纽带作用，深入开展双拥创建活动。继续做好民族宗教、人防地震、档案史志、残疾人等工作。

（七）聚焦自身建设，着力打造人民满意政府

新时代、新目标、新使命对政府工作提出了新要求。我们要把贯彻落实党的十九大精神作为首要政治任务，着力在学懂、弄通、做实上下功夫，牢牢把握精神实质，精心谋划思路举措，不断提高施政能力和推动发展水平。

打造法治政府。自觉接受人大法律监督、工作监督和政协民主监督。严格落实重大行政决策法定程序，完善决策前风险评估和合法性审查制度，认真听取法律顾问和社会各界意见建议。加强政府智库建设，建立行政决策专家咨询论证机制。全面依法履行政府职能，推进政府职能法定化和事权规范化，敬畏法纪，守住底线，做到既有为又有戒、既知行又知止。积极办理网民留言，及时回应社会关切，不断提高政府工作透明度和公信力。

打造效能政府。弘扬勇于担当、崇尚实干精神，既要有“功成不必在我”的胸怀，又要有“功成必定有我”的担当，淡化“唱功”，强化“做功”。大力精简会议文件，倡导开短会、讲短话，切实转变文风会风，把更多时间和精力用在抓落实上。提高政府执行力，定了的事，就要抓紧快办、马上就办，决不允许“磨洋工”“掉链子”。强化督查问效和效能监察，坚决克服懒政、庸政、怠政。以担当尽责为本、以干事创业为要，努力增强“八种本领”，提升广大干部服务发展能力。

打造服务政府。强化为民、惠民、便民意识，打通联系服务群众“最后一公里”，用真心真情践诺初心、温暖人心、赢得民心。把握好亲清新型政商关系，主动为企业排忧解难。把群众满意度作为评判政府工作的唯一标准，从服务群众的细微处入手、从小事做起，不断改进工作、改善服务，提升政府服务水平和治理能力。

打造廉洁政府。加强政府系统党的建设，把党的政治建设摆在首位，牢固树立“四个意识”，自觉维护以习近平同志为核心的党中央集中统一领导。扎实开展“不忘初心、牢记使命”主题教育，持续转变工作作风。严格落实中央八项规定实施细则、自治区配套实施办法和乌海市配套措施，坚决防止“四风”问题反弹。加强政务监察，强化审计监督，严肃执纪问责，零容忍惩治各类腐败行为，加大对脱贫攻坚、生态环保、土地出让等重点领域监管力度，树立风清气正、勤政廉洁良好形象。

各位代表，新时代开启新征程，新使命呼唤新作为。让我们更加紧密地团结在以习近平同志为核心的党中央周围，在自治区党委、政府和市委的坚强领导下，不忘初心、牢记使命、奋发有为，为决胜全面建成小康社会，谱写“建设亮丽内蒙古、共圆伟大中国梦”乌海新篇章而努力奋斗！
